# Characterization and Ectopic Expression of *CoWRI1*, an AP2/EREBP Domain-Containing Transcription Factor from Coconut (*Cocos nucifera* L.) Endosperm, Changes the Seeds Oil Content in Transgenic *Arabidopsis thaliana* and Rice (*Oryza sativa* L.)

**DOI:** 10.3389/fpls.2017.00063

**Published:** 2017-01-25

**Authors:** RuHao Sun, Rongjian Ye, Lingchao Gao, Lin Zhang, Rui Wang, Ting Mao, Yusheng Zheng, Dongdong Li, Yongjun Lin

**Affiliations:** ^1^Department of Bioengineering, College of Material and Chemical Engineering, Hainan UniversityHaikou, China; ^2^National Key Laboratory of Crop Genetic Improvement and National Centre of Plant Gene Research, Huazhong Agricultural UniversityWuhan, China

**Keywords:** coconut, endosperm, CoWRI1, seed-specific expression, lipid, rice (*Oryza sativa* L.)

## Abstract

Coconut (*Cocos nucifera* L.) is a key tropical crop and a member of the monocotyledonous family Arecaceae (*Palmaceae*). Few genes and related metabolic processes involved in coconut endosperm development have been investigated. In this study, a new member of the *WRI1* gene family was isolated from coconut endosperm and was named *CoWRI1*. Its transcriptional activities and interactions with the acetyl-CoA carboxylase (*BCCP2*) promoter of *CoWRI1* were confirmed by the yeast two-hybrid and yeast one-hybrid approaches, respectively. Functional characterization was carried out through seed-specific expression in *Arabidopsis* and endosperm-specific expression in rice. In transgenic *Arabidopsis*, high over-expressions of *CoWRI1* in seven independent T2 lines were detected by quantitative real-time PCR. The relative mRNA accumulation of genes encoding enzymes involved in either fatty acid biosynthesis or triacylglycerols assembly (BCCP2, KASI, MAT, ENR, FATA, and GPDH) were also assayed in mature seeds. Furthermore, lipid and fatty acids C16:0 and C18:0 significantly increased. In two homozygous T2 transgenic rice lines (G5 and G2), different *CoWRI1* expression levels were detected, but no CoWRI1 transcripts were detected in the wild type. Analyses of the seed oil content, starch content, and total protein content indicated that the two T2 transgenic lines showed a significant increase (*P* < 0.05) in seed oil content. The transgenic lines also showed a significant increase in starch content, whereas total protein content decreased significantly. Further analysis of the fatty acid composition revealed that palmitic acid (C16:0) and linolenic acid (C18:3) increased significantly in the seeds of the transgenic rice lines, but oleic acid (C18:1) levels significantly declined.

## Introduction

Coconut (*Cocos nucifera* L.), is an important tropical crop and a member of the monocotyledonous family Arecaceae (*Palmaceae*). It is the only species in the genus *Cocos* and belongs to the subfamily *Cocoideae*, which contains 27 genera and 600 species (Daniel et al., 2005). The food and industrial products derived from coconut (such as coconut oil, copra, and desiccated coconut) play an important role in the economy of many developing countries. Coconut fruit pulp (i.e., the solid endosperm obtained from mature coconuts) is the main product used for fresh consumption and/or processing. The health and nutritional benefits derived from consuming coconut oil have been recognized for centuries throughout the world (Reena and Lokesh, 2007). Although the benefits of consuming coconut oil have been well documented, the genes and metabolic pathways that make coconut oil an important functional food have remained largely unknown due to limited knowledge about their expression and related molecular biology. Endosperm development in coconut is a unique and poorly characterized process. Few genes and related metabolic processes involved in coconut endosperm development have been investigated (Knutzon et al., 1995). Therefore, there are a large number of unidentified coconut genes that could lead to the identification of new genes and functions.

The AP2/EREBP (APETALA2/ethylene-responsive element binding proteins) transcription factors, one of the largest transcription factor families in *Arabidopsis thaliana*, are only found in higher plants (Magnani et al., 2004). The members of this family have a common structural feature: all the members contain one or two highly conserved DNA binding domains (BDs) (namely the AP2/EREBP domain), which consist of about 60 amino acids (Jofuku et al., 1994; Sakuma et al., 2002). The AP2/EREBP transcription factors are found extensively in plants, and are involved in growth, development, and signal transduction in many physiological and biochemical responses, such as floral organogenesis, seed development, carbon metabolism, pathogen/stress resistance, hormone (ethylene) response, etc.

Based on the presence of the conserved AP2-like domains, 147 proteins were identified as belonging to the AP2/EREBP family in *A. thaliana* (Feng et al., 2005). The AP2/EREBP family members are classified in groups based on the number of AP2 domains and the presence of other domains. Previous research has suggested that the WRINKLED1 (WRI1)-like group is a member of the APETALA2/ethylene-responsive element binding proteins (AP2/EREBP), which share either one or two copies of a DNA-BD called the AP2 domain (Riechmann et al., 2000). The WRINKLED1 protein is an important regulator of oil accumulation in maturing *Arabidopsis* seeds. Expression of *WRI1* occurs during seed development (Cernac and Benning, 2004; Masaki et al., 2005), and the expression of genes involved in glycolysis and lipid synthesis in developing seeds is compromised in *wri1* mutants (Ruuska et al., 2002). Seeds from the wrinkled1 (*wri1*) mutant show an 80% reduction in triacylglycerols TAGs compared to the wild type (Focks and Benning, 1998).

Recently, it has been demonstrated that WRI1 is a direct target of LEAFY COTYLEDON2 (LEC2), which controls the regulatory action of the master regulator of seed maturation and fatty acid metabolism (Baud et al., 2007). The WRI1/MED15 complex transcriptionally regulates glycolysis-related and fatty acid biosynthetic genes during embryogenesis (Kim et al., 2016). It regulates a subset of genes involved in glycolysis and the incorporation of Suc into TAG (Focks and Benning, 1998). Putative targets of WRI1 encode enzymes for late glycolysis, the fatty acid synthesis pathway, and the biotin and lipoic acid biosynthetic pathways (Ruuska et al., 2002; Baud et al., 2007). The WRI1 acts as a transcriptional enhancer of genes involved in carbon metabolism in transgenic seeds over-expressing this transcription factor (Maeo et al., 2009). It has also been demonstrated that *WRI1* is able to regulate *BCCP2* and *PKp-b1* promoter activities in plants. The targets of these WRIs encode enzymes provide precursors (acyl chain and glycerol backbones) for various lipid biosynthetic pathways, but not for the subsequent lipid-assembling enzymes (To et al., 2012). Previous results have indicated that *WRI1*, which is a limiting factor on lipogenic gene expression in seeds, directly induces the transcriptional activation of these genes at the onset of the maturation phase.

Shen et al. (2010) over-expressed *ZmWRI1*, a *Wri*-like transcriptional factor from maize. This increased seed oil content by as much as 48%, which is similar to the over-expression of *ZmLEC1*, but did not affect germination, seedling growth, or grain yield. Over-expression of *ZmWRI1* increases the fatty acid content of the mature maize grain, the contents of certain amino acids, several compounds involved in amino acid biosynthesis, and two intermediates of the tricarboxylic acid cycle (Pouvreau et al., 2011). Similar attempts were also carried out in *A. thaliana* and resulted in a 10–40% increase in seed oil content, and increased seed size and mass (Adhikari et al., 2016; Kanai et al., 2016). Although *WRI1* is the best understood example of the transcription factors that have been identified in *A. thaliana* seeds, only a few have been characterized in other plants and organs/tissues (Shen et al., 2010; Pouvreau et al., 2011; Dussert et al., 2013; Ma et al., 2013) and the role of *WRI1* has never been reported in palm endosperm. In this study, a new member of the *WRI1* family was isolated, designated *CoWRI1*, and its expression patterns during fruit development analyzed. Previous research has demonstrated that that *WRI1* can be used in a seed specific manner to enhance the transcription levels of glycolytic and fatty acid biosynthetic genes in tissues where these genes are already expressed (Baud and Lepiniec, 2009). Identifying and characterizing new endosperm-specific WRI1-like transcriptional factors in these crops is very important because high quality storage of starch and proteins is essential in cereal crop endosperms.

This study shows that the ectopic expression of *CoWRI1*, driven by seed and endosperm specific promoters from *Arabidopsis* and rice, significantly increases oil content in the transgenic plant seeds. The transgenic plants showed normal growth and development without any detrimental effects on major agronomic traits. Furthermore, the expression of a subset of genes involved in fatty acid biosynthesis, glycolysis, and sugar metabolism increased in the developing seeds of the transgenic plants. This study provides a practical approach for the genetic improvement of rice and potentially other cereal crops.

## Materials and Methods

### Plant Materials and cDNA Library Construction

Coconut (*C. nucifera* L.) seeds (nut) from different developmental stages were obtained from the Institute of Coconut, Chinese Agricultural Academy of Tropical Crops (Wenchang, Hainan). Total RNA from coconut pulp in two different developmental stages was extracted by using an RNeasy kit (Tiangen, Beijing). Full-length cDNAs library were constructed according to the manual of the SMART^TM^ cDNA Library Construction Kit (Clontech, USA).

### Gene Cloning and Bioinformatics Analysis

All these clones were sequenced by the Oebiotech Co (Shanghai, PR. China). A homology search was conducted based on BLAST searches using the National Center for Biotechnology Information (NCBI) BLAST server^1^. Homology search was conducted using BLAST in GenBank^1^. Amino acid sequence analysis was performed using ORF Finder^2^. Multiple sequence alignment was performed by Clustal X 2.0 and Alignments were made in Mega 4.0.

### Quantitative Determination of Transcription Levels of CoWRI1 by RT-PCR

Total RNA from leaves as well as mature and immature coconut pulp was isolated as above. First-strand cDNA was synthesized from 2 μg of total RNA using the TIANScript OneStep RT-PCR kit (Tiangen, Beijing, China). All RT-PCR primers for the candidate genes were designed by the Primer 3 program according to the cDNA sequence. The *β-actin* gene was used as an internal control for expression. Expression was quantified in terms of comparative threshold cycle (*C*_t_) using the 2^-ΔΔCt^ method and the results were expressed as log_2_ of the relative quantity (RQ) of the normalized gene (Livak and Schmittgen, 2001). The experiment was performed in triplicate for each gene, including the no-template and no-reverse-transcriptase controls.

### Transcriptional Activation Assay of CoWRI1 Protein

The transcriptional activation activity of *CoWRI1* was identified by yeast two-hybrid analysis using *Saccharomyces cerevisiae* strain *AH109*. pGBKT7, a vector containing the *TRP1* nutritional marker for selection in yeast, and GAL4, a DNA-BD under the control of the *ADH1* promoter, were used to transform the yeast as described in the manufacturer’s instructions (Clontech, Palo Alto, CA, USA). After selection of the yeast transformants carrying the *Cowri1* gene on SD (–Trp, –Leu) medium, they were transferred to SD (–Trp, –His, –Leu, –Ade) medium to identify the transcriptional activation.

### Transcriptional Activity Investigation by Using Yeast One-Hybrid Assay

The coding region of *CoWRI1* was inserted between the *Eco*RI and *Pst*I restriction sites of the yeast expression vector pGAD-T7 containing the BD of GAL4. The reporter plasmid was constructed by inserting fragments of the *BCCP2* promoter into the pHIS2.1 vector according to Baud and Lepiniec (2009). These two plasmids were introduced into the yeast strain Y187 with the reporter gene *His3* by the same method as described for the DRE-binding analysis. If the encoded proteins possessed activation ability, it would work together with the BD of GAL4 to promote the expression of the reporter gene *His3*, resulting in the growth of the transformed yeast cells on the SD/-His + 10 mM 3-AT medium. Yeast cells containing pGAL-T7 was used for the negative controls (Baud and Lepiniec, 2009).

### Plasmid Construction for Plant Transformation

To construct plant expression vectors harboring *CoWRI1* genes, entirely ORF of *CoWRI1* gene were inserted into pCAMBIA1300S using *Bam*HI and *Pst*I sites, leading to pCAMBIA1300S- CoWRI1. The upstream CaMV35S promoter was replaced by an endosperm-specific promoter *EnP2* from rice (Chinese patent, ZL201010146054.0), generating fusion between the *EnP2* promoter and the *CoWRI1* cDNA. The related molecular procedures, such as fragment purification, ligation, and transformation, were performed as previously described Sambrook et al. (1989). Plasmid construction and manipulation was carried out according to the previously described standard methods. The constructed vectors were verified by PCR and sequencing.

### Generation of Transgenic Plants

The obtained expression vectors were sequenced to verify the gene orientation and transferred into *Agrobacterium tumefaciens EHA105* by a freeze–thaw method (Huang et al., 2001). *Arabidopsis* were transformed by the floral dip method (Clough and Bent, 1998). Transformants were selected with hygromycin (*hyg*) resistance and confirmed by PCR using primers of *hyg* (**Table 1**). Wild type *Arabidopsis* were grown in separate flats in the same incubator. All plants were individually tied to stakes. For rice transformation, the constructs were introduced into Zhonghua11 (*Oryza sativa* L. ssp. *japonica*) by *Agrobacterium*-mediated transformation. The callus culture and transformation procedures were carried out as in Hiei et al. (1994). The putative transgenic plants obtained from all experiments were validated by PCR and Southern blot analysis (Lin and Zhang, 2005). Only containing single-copy plants without obvious phenotype change were bagged for the production of self-pollinated T_1_ seeds. The subsequently surviving T_1_ seedlings were transferred to soil to set T_2_ seeds. T_2_ seeds and seedlings were used for the following analysis.

**Table 1:** Sequence of all primers.

Primer	Forward	Reverse
CoWRI1	*ACGGATCCATGACCCTCATGAAGAAGAAG*	*AGCTGCAGCTAGGCATCCTTTGTTGCACT*
*WRI1*	*TTCCTTTTCCGCTTCACC*	*CTTCATACCTTCCAGTCCCTC*
*Actin7*	*GCCCCTGAGGAGCACCCAGTT*	*CCGGTTGTACGACCACTGGCA*
GAPDH	*CCCTTCATCACCACCGACTA*	*CCCTCAACAATACCAAACCTG*
*PKp2*	*AGTCACTATCGTCCTTCCG*	*CTGTACGATTGCTATTTCCTC*
*MAT*	*CATGGTTAGTATCATAGGGTTGGACTCAGA*	*TGGCTTCAACAACTTCGATTCCTTTAAGA*
*KAS1*	*GGGTTCTGCTTTGTTGGCGA*	*GCCTCAGTCCCACCAGCAAT*
*KASIII*	*TCTGTGGCTACAAGGCTGCAT*	*TGCTGATCCCCACGTTAAACCG*
*BCCP2*	*GACCCGGTGAACCCCCT*	*GTCAACGCTGACTGGTTTTCCAT*
*ENR*	*TGGGACTTGGGTTCCTGCAC*	*CGCTTATTCGTTTTCACATCTTCAGGC*
*FATA*	*ACAACAACTACTATGAGGAAGTTGCATCT*	*TCCAATCACGCCTTGTCCC*
*GPDH*	*TGTGGAAGCAGAGTTTGAGCCT*	*ACCCGCAAATCCGTGCAT*
*GPDHc1*	*GGGAGGTCTCAAGAATGTCTACGC*	*AGCAAAGGCCCTGCAAGTT*
*GPAT9*	*GCCTCATGGACCGGAGTTGT*	*CCAACCCAACCAGGATGCT*
*Hyg*	*GGACTTCGGGGCAGTCCTT*	*CGATGTAGGAGGGCGTGG*

### Analysis of Gene Expression by qRT-PCR

Total RNA was prepared using Trizol methods (Invitrogen, Carlsbad, CA, USA). The first-strand cDNA was synthesis by SuperScript II reverse transcriptase using oligo (dT) as a primer (Invitrogen). Reverse transcriptional products were used as a template for real-time PCR and *actin7* of *Arabidopsis* and *GAPDH* gene in rice as inner control separately. The RT-PCR amplification step was performed using the SYBR^®^ Premix Ex Taq^TM^ II (TaKaRa) and a RT-PCR detector (TaKaRa Smart Cycler II system) by using the SYBR Green I chimeric fluorescence method according to the manufacturer’s instruction. Reactions were performed in triplicate, including the no-template and no-reverse-transcriptase controls, and were monitored using an Applied Biosystems 7500 RT-PCR instrumentation outfitted with SDS software version 1.3.1.

### Analysis of Starch, Protein, and Fatty Acid Content

Samples were ground to a fine powder in liquid nitrogen and then transferred to a plastic centrifuge tube. Starch contents were analyzed as described by Lunn and Hatch (1995). Protein contents were determined and analyzed as described by Sedmak and Grossberg (1977). Total seed oil was extracted using dichloromethane/methanol (2:1) from mature seeds from each of the transgenic lines and WT seeds of rice, solidified under nitrogen gas ventilation, and transmethylated with 2% KOH-methanol (m/v) at 80°C for 2 h. The fatty acid methyl esters (FAMEs) were recovered using *n*-hexane. Analysis of FAMEs was performed using GC, with mixed fatty acid methyl (C_8_–C_22_) (CRM18920, Sigma) as standard. All GC analysis was performed using a HP5890 GC instrument equipped with a BPX-70 (30 m × 0.25 mm) chromatography column. All experiments were carried out in three sets of parallel experiments.

## Results

### Isolation and Structural Analysis of CoWRI1

**FIGURE 1:**
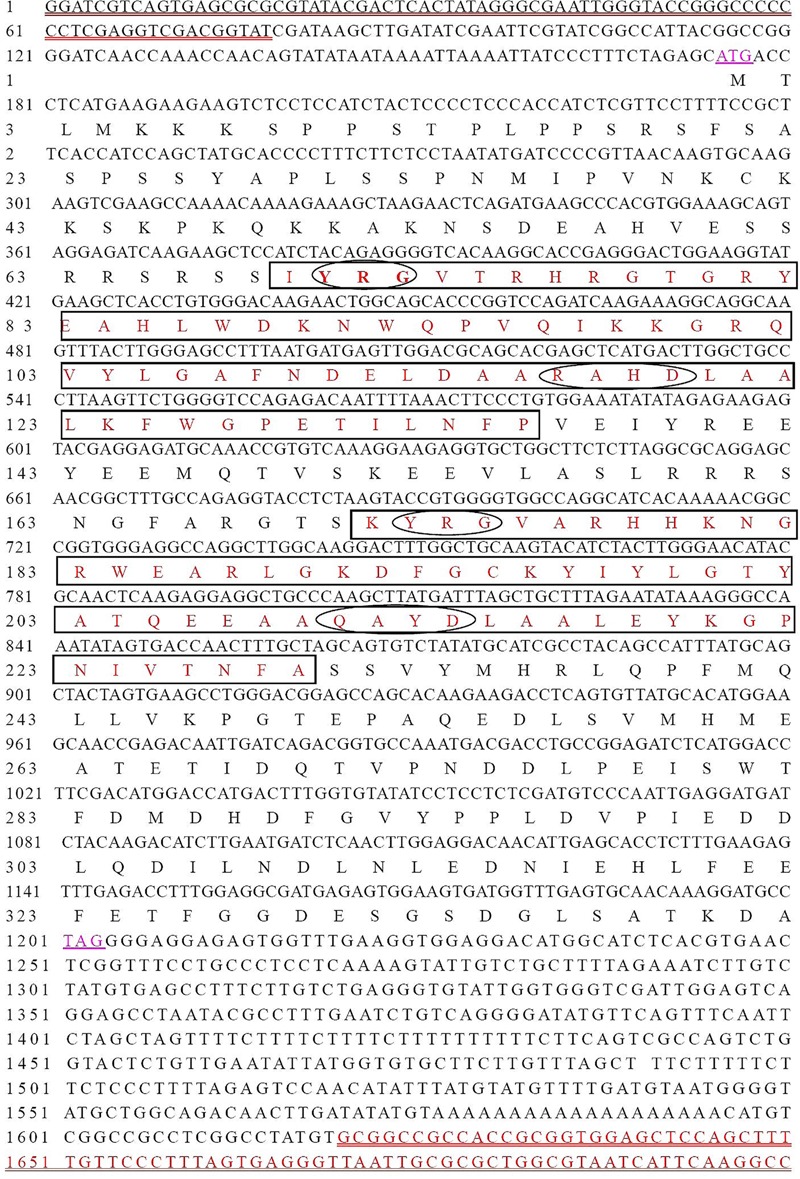
**Deduced amino acid sequences of CoWRI1 proteins and conserved domain analysis.** The two rectangular frames indicate the two conserved DNA-binding domains (BDs) (AP2/ERF domain), and the elliptical frame indicates BDs. The double underlined sequence is part of the vector.

**FIGURE 2:**
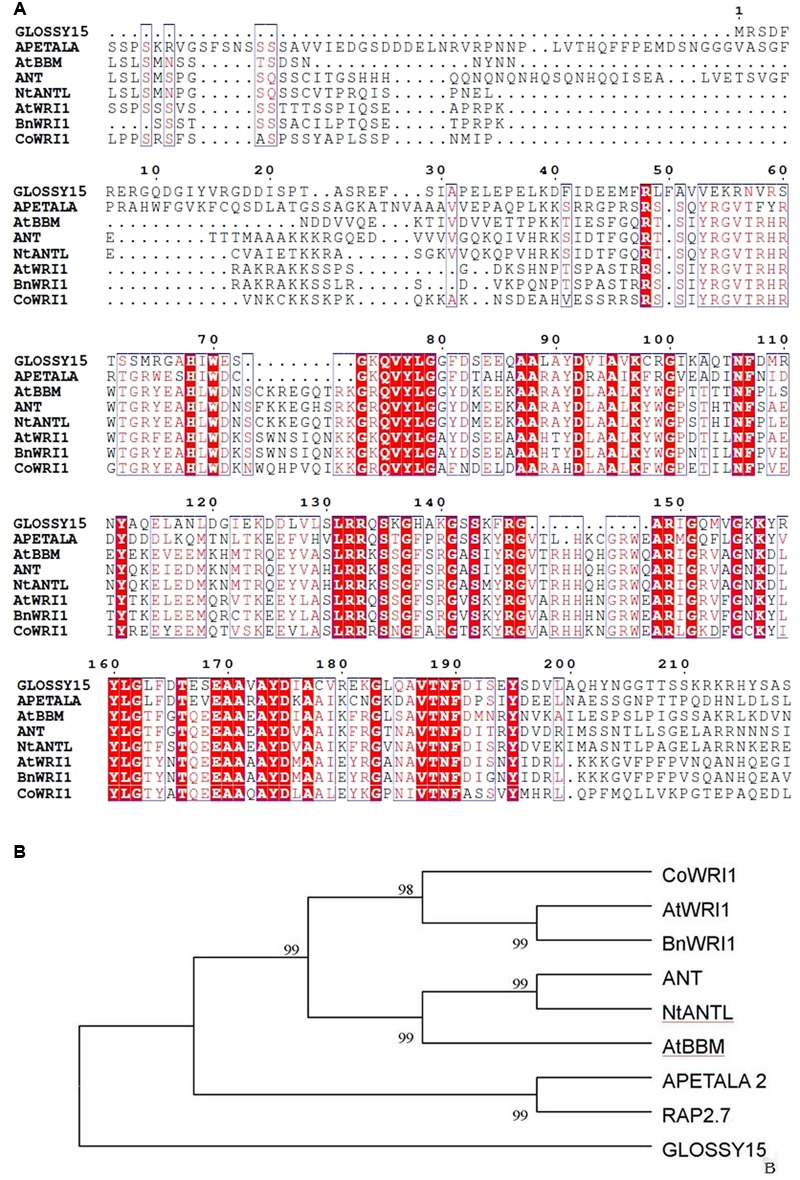
**(A)** Comparison of the deduced amino acid sequences for AP2/ERF-related proteins that have high sequence similarity with CoWRI1. Amino acid residues that are conserved in at least three of the nine sequences are in black, whereas amino acids that have at least six identical proteins are shown in red. **(B)** Phylogenic comparison of the CoWRI1 protein and some AP2/ERF-related protein sequences based on the selected AP2/ERF domain amino acid sequences for those proteins.

The full-length *CoWRI1* clone (GenBank Accession No. AFH68065.1) is 1,051 bp in size. It includes an open reading frame of 1,029 bp and encodes a putative protein of 342 amino acids with a predicted molecular mass of 38.79 kDa and a pI of 5.82. Analysis of the deduced amino acid sequence revealed that this protein had two typical AP2/ERF DNA-BDs at 69–135 aa and 171–229 aa. It showed a high sequence identity with the AP2-ERF domains of other members of the AP2 family. Furthermore, CoWRI1 has conserved YRG and RAYD residues in two AP2/ERF domains (**Figure 1**), which suggests that CoWRI1 is likely to be a member of the ERF subfamily among the AP2/EREBP proteins. The sequence alignment results (**Figure 2A**) showed that CoWRI1 has limited sequence identities with APETALA2 (GI: 30690802), AtBBM (GI: 151936653), AtWRI1 (GI: 145339487), ANT (GI: 30691332), and RAP2.7 (GI: 145360425) from *A. thaliana*; NtANTL (GI: 38492171) from *Nicotiana tabacum*, BnWRI1 (GI: 87042569) from *Brassica napus* and EgWRI1 (GI:615794066) form *Elaeis guineensis* which are all members of the AP2/EREBP subfamily in the database. It showed high open reading fragment homology at the amino acid level with two conserved AP2/ERF domains, which suggests that CoWRI1 has a similar function to other previously identified WRI1 like proteins. Alignment and phylogenetic tree analysis revealed that CoWRI1 is similar to AtWRI1 and BnWRI1, which are ERF and WRI1 subgroup members (Sakuma et al., 2002) (**Figure 2B**). CoWRI1 has 59–80% overall amino acid sequence identity with *E. guineensis* EgWRI1, *A. thaliana* WRI1, *B. napus* WRINKLED, and *Zea mays* AP2/EREBP transcriptional factor WRI1.

### Expression Analysis of CoWRI1 in Different Tissues

**FIGURE 3:**
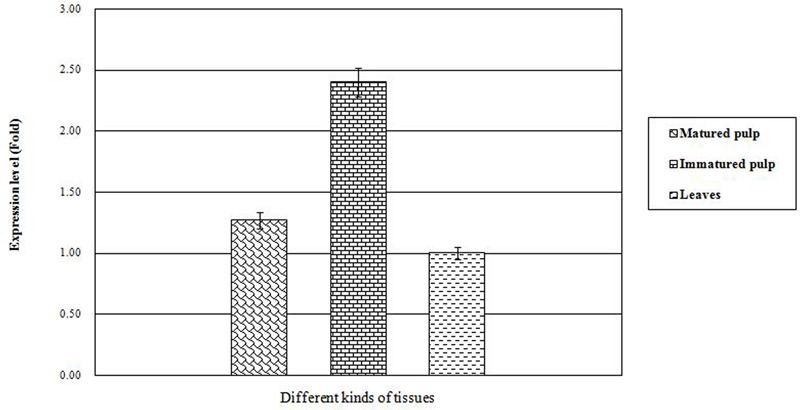
**Expression patterns for CoWRI1 in different tissues**.

In order to reveal the expression patterns of *CoWRI1* in coconut, quantitative Real-time PCR (qRT-PCR) was performed to examine the transcription levels of *CoWRI1* genes in the leaves, mature endosperm (12-months-old) and immature endosperm (8-months-old). The results demonstrated that *CoWRI1* was highly expressed in immature endosperm, whereas its expression was lower in mature endosperm and young leaves (**Figure 3**). The immature endosperm is the most active stage of fatty acid anabolism, and these results are consistent with previous estimations.

### CoWRI1 Transcriptional Activation Activity

**FIGURE 4:**
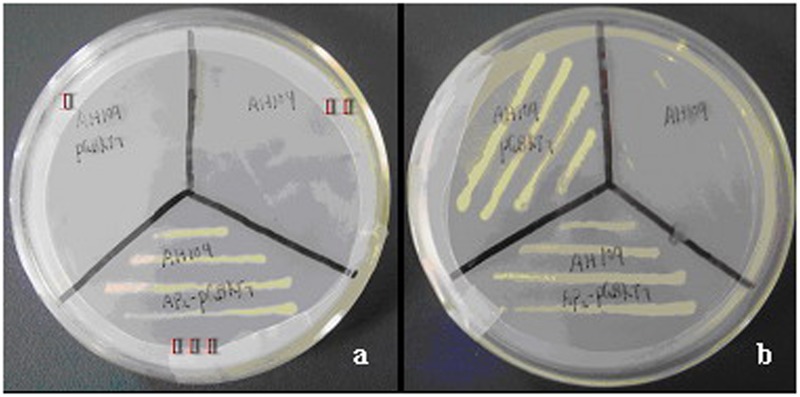
**Transcriptional activation activity of CoWRI1.** Empty AH109 (II), AH109 harboring pGBKT7 (I), and AH109 harboring pGBKT7-CoWRI1 (III) were grown on selective mediums. **(a)** SD/-Trp/-Ade/-His and **(b)** SD/-Trp.

Yeast two-hybrid analysis was used to determine whether *CoWRI1* could act as a transcriptional activator in yeast. The full-length *CoWRI1* gene was fused to the DNA-BD of GAL4 (Clontech, Palo Alto, CA, USA) to identify the transcriptional activation activity by growing the yeast cells on SD/–Trp and SD/–Ade/–His/–Trp media. Yeast cells carrying the pGBKT7 plasmid, which contains only the GAL4 DNA-BD, were used as the negative control because they can grow on the SD/–Trp substrate, but not on the SD/–Ade/–His/–Trp medium. The results indicated that when the GAL4 activation domain is present, yeast cells carrying full-length *CoWRI1* fused to the GAL4 DNA BD activated the transcription of downstream reporter genes and allowed the yeast to grow on SD/–Ade/–His/–Trp medium (**Figure 4**).

### Interaction of WRI1 with the BCCP2 Promoter

**FIGURE 5:**
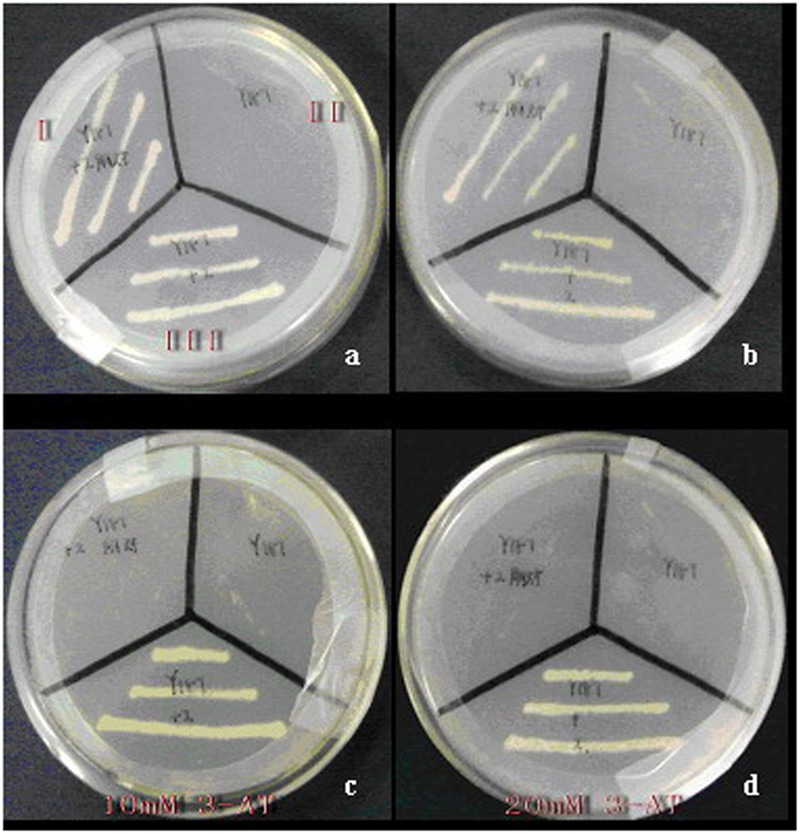
**Interaction between WRI1 and the BCCP2 promoter.** Empty Y187 (II), Y187 harboring pGADT7-AD, pHIS2.1-BCCP (I) and Y187 harboring pGADT7-AD-CoWRI1, and pHIS2.1-BCCP2 (III) were cultured on different selective mediums. **(a)** SD/-Trp/-Leu; **(b)** SD/-Trp/-Leu/-His; **(c)** SD/-Trp/-Leu/-His containing 10 mM 3-AT; **(d)** SD/-Trp/-Leu/-His containing 20 mM 3-AT.

No target gene for *CoWRI1* was identified in coconut, and *CoWRI1* shared a highly conserved domain with *AtWRI1* (from *A. thaliana*) and *BnWRI1* (from *B. napus*). The *BCCP2* promoter sequence was amplified from the *A. thaliana* genome and fused into pHIS2.1, which resulted in pHIS2.1-BCCP2. A yeast one-hybrid approach was used to analyze the interaction between *CoWRI1* and the *BCCP2* promoter. The transformation of the strains presenting the *HIS2.1* reporter gene under the control of *BCCP2* promoters and pGADT7-AD produced no positive interaction results. However, the expression of *CoWRI1* fused to pGADT7-AD in the strain presenting the *HIS2.1* reporter gene under the control of the *BCCP2* promoter resulted in the specific growth of the strain on a medium lacking histidine (His), leucine (Leu), and tryptophan (Trp), which showed that there was an interaction between *WRI1* and this promoter sequence. Further analysis suggested that yeast cells carrying the full-length *CoWRI1* can also grow on a SD/–His/–Leu/–Trp medium containing 10 mM 3-amino-1, 2, 4-triazole (3-AT) or 20 mM 3-AT (**Figure 5**).

### Generation of Transgenic *Arabidopsis* and Gene Expression Analysis by qRT-PCR

**FIGURE 6:**
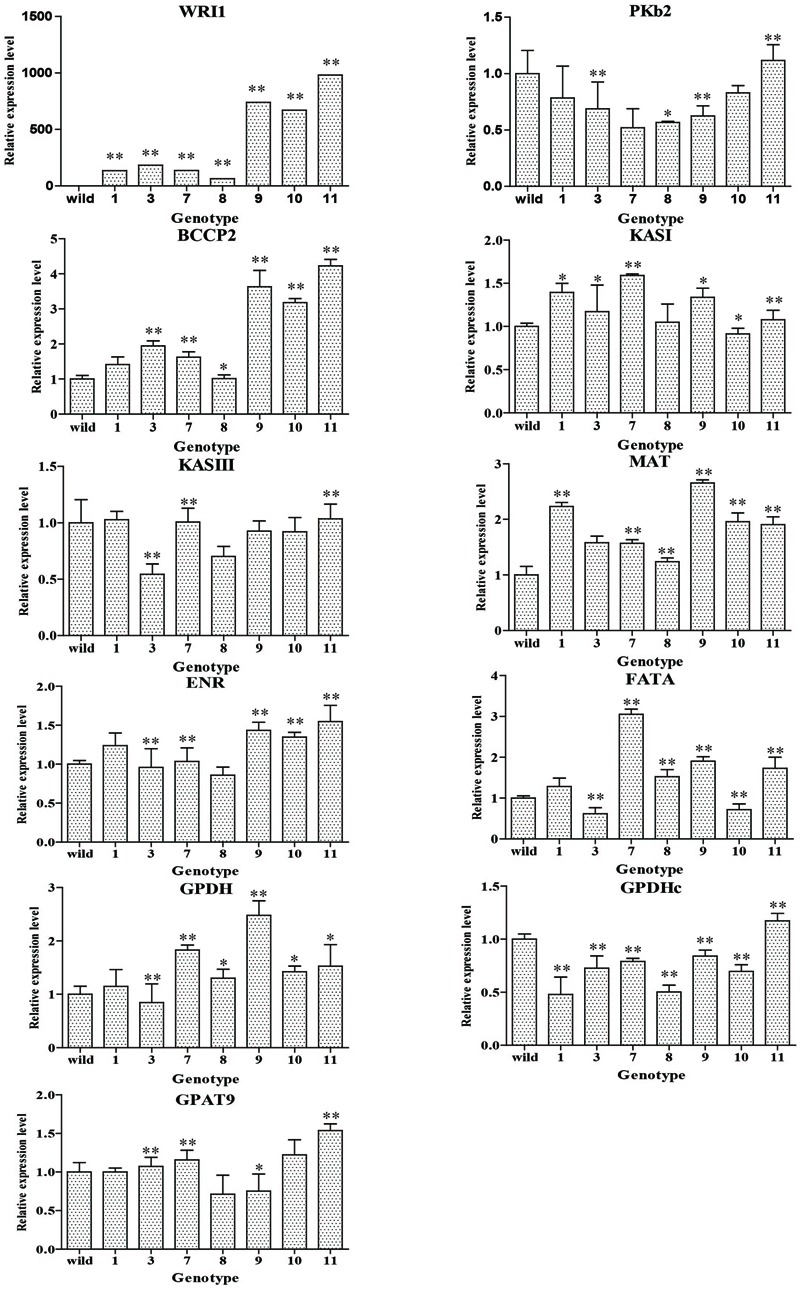
**Expression analysis of *Cowri1* and genes involved in fatty acid (FA) biosynthesis in mature seeds from transgenic *Arabidopsis thaliana*.** Wild, the wild type (Col-0). BCCP, acetyl-CoA carboxylase (At5g15530); ENR, enoyl-ACP reductase (At2g05990); FAT, fatty acyl-ACP thioesterase (At2g30200); G3PDH, glycerol-3-phosphate dehydrogenase (At2g41540 and At5g40610); GPAT, glycerol-3-phosphate acyltransferase (At5g60620); KAS, 3-ketoacyl-ACP synthase (At5g46290 and At1g62640); MAT, malonyl-CoA: ACP transacylase (At2g30200); PKp, plastidial pyruvate kinase (AT1G32440). ^∗^Significant difference according to Student’s *t*-test at *P* < 0.05. ^∗∗^Extremely significantly different, *P* < 0.01.

The plant expression vectors harboring *CoWRI1* driven by a seed-specific promoter were sequenced and transferred into *A. tumefaciens EHA105* for *Arabidopsis* transformation. Fifteen independent transgenic plants were obtained after gene transformation and plant regeneration. Subsequently, eight positive transformants were revealed by PCR analysis. These positive transformants were self-pollinated to produce T_1_ lines and 20 T_1_ transgenic plants for each positive family were grown for seed collection. The mature seeds from the selected T_1_ transgenic plants were collected separately. After a seed germination test, homozygous transgenic lines from each family were selected for further analysis. *CoWRI1* expressions in seven independent T_2_ transgenic lines were analyzed by qRT-PCR. Different *CoWRI1* expression levels were detected in the seven transgenic lines, but no *CoWRI1* transcripts were detected in the negative control. Among the seven transgenic lines, WRI1-11 showed the highest expression level, which was about 15.34-fold higher than line WRI1-8 (**Figure 6**). To further elucidate the changes in seeds that over-expressed *CoWRI1*, the qRT-PCR approach was also used to systematically analyze the relative mRNA accumulation for genes encoding enzymes involved in either fatty acid biosynthesis or TAG assembly. These included *PKp* (plastidial pyruvate kinase), *MAT* (malonyl-CoA: ACP transacylase), *BCCP2* (BIOTIN CARBOXYL CARRIER PROTEIN2), *KAS* (3-ketoacyl-ACP synthase), *ENR* (enoyl-ACP reductase), *FAT* (fatty acyl-ACP thioesterase), *GPDH* (glycerol-3-phosphate dehydrogenase), and *GPAT* (glycerol-3-phosphate acyltransferase) (**Figure 6**). The results indicated that seed-specific expression of *CoWRI1* in *A. thaliana* up-regulated genes that are involved in fatty acid synthesis by varying degrees (**Figure 6**).

In the three highly over-expressing homozygous transgenic lines (lines WRI1-9, WRI1-10, and WRI1-11), some of the glycolytic and late fatty acid biosynthetic genes (*BCCP2, MAT, ENR*, and *GPDH*) were significantly up-regulated in mature seeds, but the expression levels in the other lines did not significantly increase or even declined. The *BCCP2, ENR*, and *GPDH* effects were synchronized in the seven transgenic lines, but other gene effects were not. This was most apparent in the highest over-expression line (WRI1-11). Almost all the genes were involved in fatty acid biosynthesis and TAG assembly. This was particularly the case for line WRI1-11, which showed the highest over expression.

### Analysis of Seed Lipid and Fatty Acid Contents in Transgenic *Arabidopsis*

**FIGURE 7:**
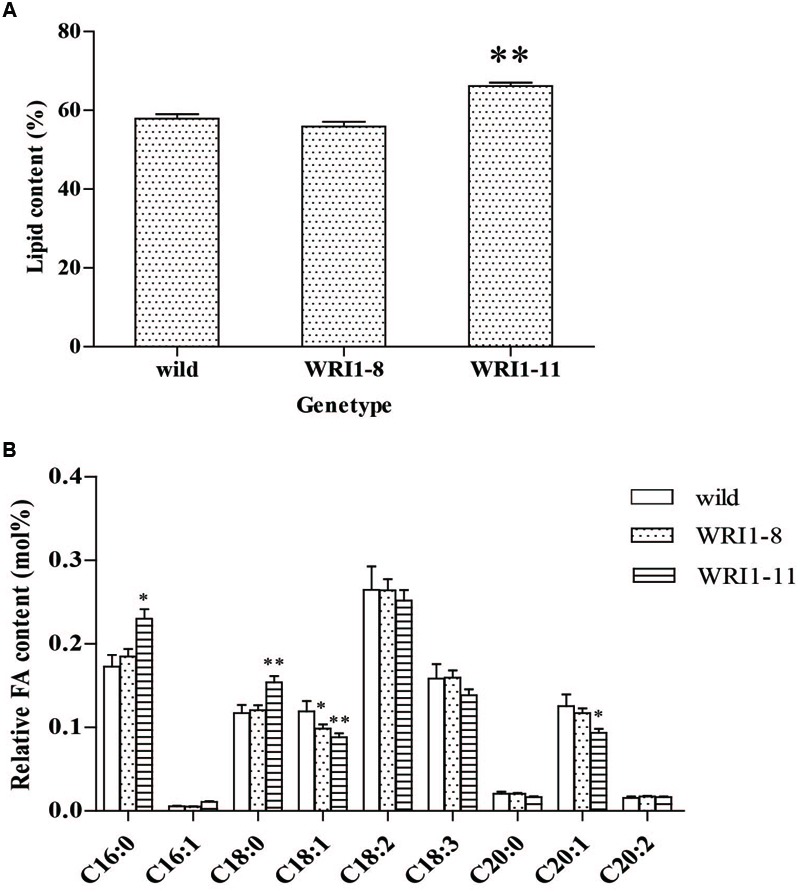
**Lipid and relative fatty acid contents of mature *A. thaliana* seeds. (A)** Lipid content of wild type (wild), line WRI1-8 and line WRI1-11. Values are the means and SD of three replicates of dry mature seeds. ^∗∗^Significant difference according to *t-*test, *P* < 0.01. **(B)** Relative FA content in mature seeds following seed-specific overexpression of WRI1 cDNA. Wild, wild type(Col-0). ^∗^Significant difference according to Student’s *t*-test at *P* < 0.05. ^∗∗^Extremely significantly different, *P* < 0.01.

Two independent T_2_ transgenic lines from the *A. thaliana* lines with the highest and lowest over-expression levels were analyzed for the seed lipid content in order to observe the effects of *CoWRI1* over expression. The analysis of seed lipid content in the representative transgenic lines indicated that the WRI1-11 line (the highest) significantly increased (*P* < 0.01), whereas the WRI1-8 line (the lowest) showed no significant change (**Figure 7A**). Further analysis of fatty acid composition using gas chromatography-mass spectrometry (GC-MS) revealed that palmitic acid (C16:0) and stearic acid (C18:0) increased significantly in WRI1-11 transgenic line seeds compared to the wild type plants. The oleic acid (C18:1) and eicosanoic acid (C20:1) levels significantly decreased, whereas the acid levels in the C16:1 seeds did not change significantly. However, in the WRI1-8 transgenic line, only oleic acid (C18:1) significantly decreased, whereas the other fatty acids did not significantly change (**Figure 7B**).

### Nutrient Analysis and Ectopic Expression of CoWRI1 in Rice Seeds

**FIGURE 8:**
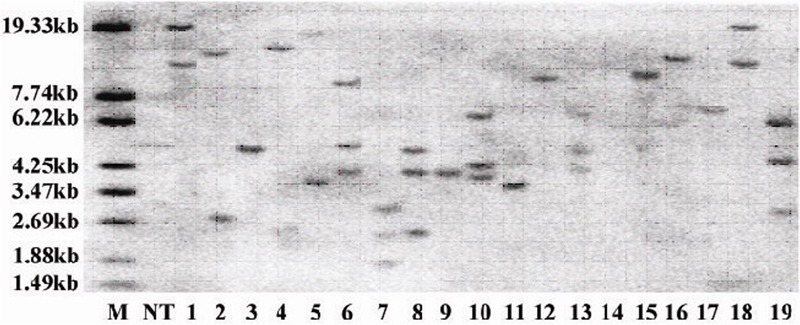
**Southern blot analysis of the total genomic DNA from T_0_ transgenic plants.** The genomic DNA samples were digested with *Hind*III and hybridized with a prepared radioactive probe. M: DNA marker; NT: Zhonghua 11; lanes 1–19, transgenic plants.

*CoWRI1* was ectopically applied to rice endosperm in order to evaluate whether *CoWRI1* has any potential application value when attempting to genetically improve crops. Thirty-one independent transgenic plants were obtained after gene transformation and plant regeneration, and 28 transgene-positive transformants were revealed by PCR analysis. These positive transformants were further confirmed by Southern blot analysis (**Figure 8**). Eight single-copy insertion plants were transferred to the field for trait detection. Two single-copy insertion plants that did not show any significant phenotypic changes were self-pollinated to produce T_1_ lines. Twenty T_1_ transgenic plants from two single-copy families were grown in the field. The mature seeds from selected T_1_ transgenic plants were collected separately and used for a germination assay. After the seed germination test, one of the homozygous transgenic lines from each single-copy family was selected for further analysis. The selected two homozygous transgenic rice lines were named G2 and G5.

**FIGURE 9:**
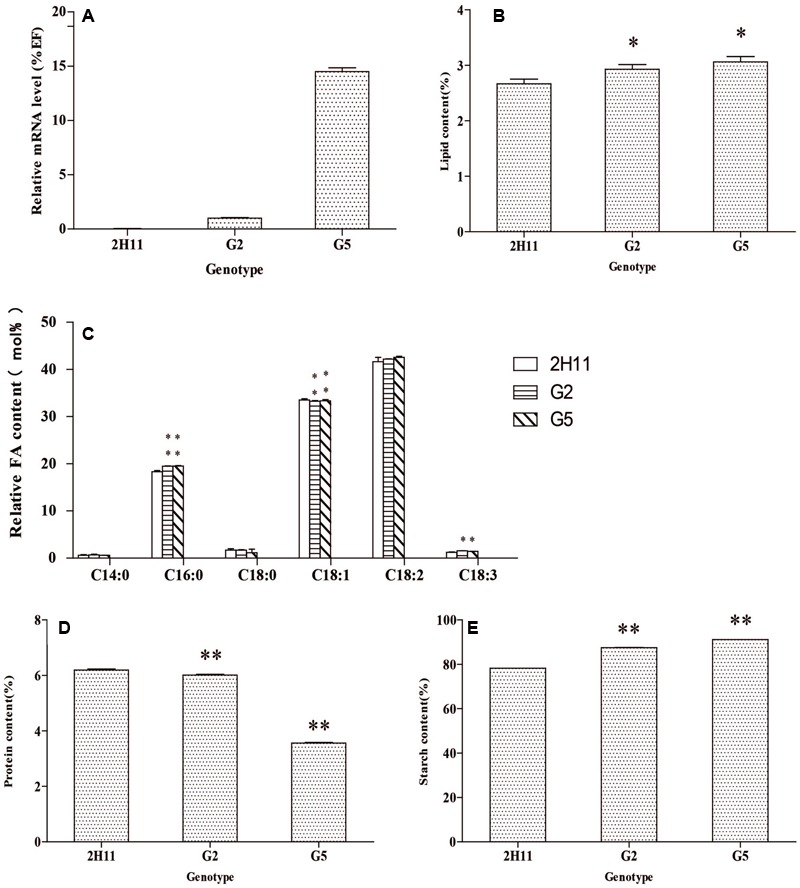
**Analysis of the seed oil content, starch, and total protein in two different T_2_ transgenic lines. (A)** Expression analysis of *CoWRI1*in three different transgenic lines. **(B)** The oil contents in four different transgenic lines. **(C)** Fatty acid composition analysis of three transgenic lines by gas chromatography-mass spectrometry (GC-MS). **(D)** Total protein content in three different transgenic lines. **(E)** The percent starch content of three different transgenic lines. ZH11 was used as the control. Bars indicate SD (*n* = 3). Asterisks indicate a significant difference from control (ZH11). (Student’s *t*-test: ^∗^*P* < 0.05 and ^∗∗^*P* < 0.01).

The *CoWRI1* expressions in two independent T_2_ transgenic rice plants were analyzed by real-time PCR. Different *CoWRI1* expression levels were detected in three transgenic lines, whereas no *CoWRI1* transcripts were detected in the control. G5 showed the highest expression level, which was about 14.5-fold higher than G2 (**Figure 9A**). Each independent T_2_ transgenic line was analyzed for seed oil, starch, and total protein content in order to observe the effects of *CoWRI1* over-expression. Analysis of seed oil content by the Soxhlet extraction method in the representative transgenic lines indicated that seed oil content had significantly increased (*P* < 0.05) (**Figure 9B**). Total fatty acids were analyzed by GC-MS in three sets of parallel experiments. The results indicated that palmitic acid (C16:0) and linolenic acid (C18:3) levels in the transgenic seeds were significantly higher than in the wild type seeds. The oleic acid (C18:1) level significantly decreased, whereas the C14:0, C18:0, and C18:2 contents did not significantly change (**Figure 9C**). Starch content analysis revealed that both transgenic lines had significantly higher starch contents (*P* 0.01) (**Figure 9E**). However, the total protein content quantitative analysis showed that both transgenic lines had significantly lower total protein contents (*P* <0.01) (**Figure 9D**). Endosperm-specific expression of CoWRI1 reduced protein content in the endosperm, but increased oil and starch contents, which suggested that CoWRI1 may enhance oil biosynthesis by increasing carbon flux to starch biosynthesis in transgenic lines.

## Discussion

The *WRI1* transcription factor was the first positive regulator of fatty acid biosynthesis identified in plants (Cernac and Benning, 2004). Furthermore, conditional over-expression of *WRI1* does not bring undesirable agronomic traits, such as poor germination and plant growth. This means that the gene has a high potential crop genetic improvement value, especially with regards to raising fatty acid/lipid contents. To date, WRIs are the only known transcriptional regulators of de novo fatty acid synthesis in plants (To et al., 2012). In this study, one WRI1-like transcription factor was identified from coconut endosperm and its function was investigated by conditional heterologous expression in the endosperm of transgenic rice plantlets. To our knowledge, this is the first report on the cloning and functional analysis of the *wri1*-like transcriptional factor from a palmaceous plant species that was characterized in and used for cereal genetic modification. The results also confirmed that this kind of regulatory element might be conserved between different plants and can thus be used in a number of different crops.

A previous study on the co-expression of *WRI1* reported that, on average, there was a 20- to 140-fold trans-activation of *Pl-PKb1* and *BCCP2*, and a 5- to 10-fold trans-activation of *KAS1* and *ACP1* genes. The co-expression of WRI1 also showed a 5- to 10-fold trans-activation of the upstream sequences in *CWINV2* and *SUS2* (Maeo et al., 2009). However, in the transgenic plants that over expressed *CoWRI1* from coconut, similar trans-activation effects were only just detected for *BCCP*, but not *PKb1* and *KASI*. This may be because the samples collected for qRT-PCR in this study were mature seeds and the mRNA levels of the above-mentioned genes decline in the late seed maturation stage. The *FATA* gene, which showed the greatest accumulation of mRNA, slightly lagged behind the above mentioned genes and its levels remained high during the later seed maturation stages. The significance of this increased *FATA* expression would mean with respect to improvement of oil production in transgenic plants.

The *CoWRI1* over-expression effect on lipid content in transgenic *Arabidopsis* (*P* < 0.01) and rice (*P* < 0.05) seeds were different, although the gene expression levels were very high in both of them. Moreover, the impact on fatty acid content in transgenic *Arabidopsis* seeds [palmitic acid (C16:0) and stearic acid (C18:0) increased significantly] significantly differed from the effect in transgenic rice seeds [palmitic acid (C16:0) and linolenic acid (C18:3) were significantly increased]. The results suggested that *CoWRI1* expression in specific tissues and the change in lipid content were mainly based on the original lipid synthesis levels (about 2% in rice seed and 35% in *Arabidopsis* seeds). Previous studies also suggested that factors related to seed lipid content correlated with the presence of fatty acid synthesis and metabolic pathways, and was also closely related to the tricarboxylic acid cycle, the pentose phosphate pathway, and glycolysis (Bourgis et al., 2011). The over-expression of *CoWRI1* only affected lipid content and the types of fatty acids species produced, but together with over-expression of plastid carbon supply related genes is likely more crucial for the eventual accumulation of oil in transgenic plant seeds.

The lipid fraction in the rice (*O. sativa* L.) grain is stored as TAG in the oil bodies and is largely concentrated in embryos and the aleurone layers of the endosperm (Takemoto et al., 2002). Its biological function is to supply a ready source of energy to the germinating grain, and its nutritional benefit to the human diet has been well recognized. Five major fatty acids have been characterized in brown rice (16:0, 18:0, 18:1, 18:2, and 18:3) and some minor fatty acids have also been identified. However, there are substantial variations among rice cultivars. Previous studies indicated that lipid content has a marked influence on rice appearance and the eating quality of cooked rice, and that change in lipid composition had important effects on rice aging and deterioration (Thucle et al., 2013). Most of the quality *indica* cultivars in China have rice grains with high lipid content, and show good glossiness, palatability, and fragrance (Shen et al., 2012). Lipid content also affects the processing and cooking quality of rice. Thus, finding an efficient strategy to improve the lipid content of rice grains in order to breed new varieties of rice is very important.

Like many economically important traits, rice lipid content is quantitatively inherited. The identification of quantitative trait loci (QTLs) for lipid content was considered essential for the development of marker-assisted selection strategies to improve the lipid content of brown rice. Therefore, a number of QTLs involved in fatty acid composition and oil concentration have been identified (Liu et al., 2010). Although the QTLs controlling oil content in brown rice have been identified (Ying et al., 2012), few or no QTL analyses have been carried out for fatty acid composition in the monocot model plant to date. The *WRI1* transcription factor is the first positive regulator of fatty acid biosynthesis identified in plants and could be a promising method for improving major crop genetics and breeding. However, the tissue specific and conditional expression of the *WRI1* transcription factor should be strictly controlled so that the increase in oil content does not produce undesirable changes in seed germination and crop growth.

In a previous study, Cernac and Benning (2004) reported that the expression of *WRI1* cDNA under the control of the cauliflower mosaic virus 35S promoter led to a slight increase in seed oil content. However, *Pro35Sdual:WRI1* and *ProS2:WRI1* transgene expressions in a wild-type background (Baud and Lepiniec, 2009) or the introduction of *ProAT2S2:WRI1* in a wri1-4 mutant background led to efficient accumulation of oil in the corresponding transgenic seeds. Shen et al. (2010) showed that *ZmWRI1*, under the control of an embryo-preferred promoter, substantially increased the oil content of the seed. However, expression under the control of the 19KD *ZEIN* promoter in maize endosperm did not cause a significant increase in seed oil content. This is because maize endosperm consists of a central mass of starchy endosperm cells, a single layer of aleurone cells surrounding the starchy endosperm, and a basal layer of transfer cells, but only the aleurone cells accumulate oil, whereas starch endosperm cells do not (Olsen, 2001). Therefore, the failure of *ZmWRI1* to increase oil in the starchy endosperm of maize was because the genes involved in oil biosynthesis and oil body formation were not expressed. In this study, endosperm-specific promoter *EnP2* was used to conditionally express *CoWRI1* in the endosperm of *O. sativa* L. ssp. *Japonica* rice, and a significant increase of fatty acid was detected in the obtained transgenic seeds. It contrast to the starch endosperm of maize, rice endosperm contains multiple genes for fatty acid and oil biosynthesis. Consequently, starch in the endosperm of transgenic rice was efficiently converted into oil by tissue specific over-expression of *CoWRI1*. Starch endosperm in rice accounts for about 90% of seed mass, which means that the conversion of starch to oil in starchy endosperm cells will considerably increase seed oil content.

## Conclusion

The aim of this study was to characterize a new member of the WRI1 family that had been isolated from coconut endosperm. The gene was named *CoWRI1*. Functional analysis was carried out by conditionally expressing it in transgenic *Arabidopsis* and rice seeds under a seed-specific and endosperm-specific promoter. Seed-specific over-expression of *CoWRI1* in *A. thaliana* up-regulated genes involved in fatty acid synthesis to varying degrees, and there was a significant increase in seed lipid content. The ectopic expression of *CoWRI1* in transgenic rice also produced a significant increase in seed oil content and total starch content, but led to a reduction in protein content. The fatty acid composition analysis revealed that palmitic acid (C16:0) and linolenic acid (C18:3) increased significantly in the seeds produced by three transgenic lines, but oleic acid (C18:1) levels significantly declined. The endosperm-specific AP2/EREBP domain-containing transcription factor created from coconut in this study could be used in future crop genetic research.

## Author Contributions

DL and YL designed the research. RS, RY, LG, LZ, RW, TM, and YZ performed the research. RS and DL wrote the paper. All authors read and approved the final manuscript.

## Conflict of Interest Statement

The authors declare that the research was conducted in the absence of any commercial or financial relationships that could be construed as a potential conflict of interest.

## Footnotes

1 http://www.ncbi.nlm.nih.gov/BLAST

2 http://www.ncbi.nlm.nih.gov/gorf/gorf.html
